# DNA Holliday Junction: History, Regulation and Bioactivity

**DOI:** 10.3390/ijms23179730

**Published:** 2022-08-27

**Authors:** Qinqin Song, Yuemiao Hu, Anqi Yin, Hongbo Wang, Qikun Yin

**Affiliations:** 1 State/Key Laboratory of Microbial Technology, Shandong University, 72 Jimo Binhai Road, Qingdao 266237, China; 2 Key Laboratory of Molecular Pharmacology and Drug Evaluation, Ministry of Education, Collaborative Innovation Center of Advanced Drug Delivery System and Biotech Drugs in Universities of Shandong, School of Pharmacy, Yantai University, 30 Qingquan Road, Yantai 264005, China; 3 Bohai Rim Advanced Research Institute for Drug Discovery, 198 Binhai East Road, Yantai 264005, China

**Keywords:** DNA Holliday junction, HJ nuclease, DNA damage, chromosomal aberration, HJ-targeting ligand

## Abstract

DNA Holliday junction (HJ) is a four-way stranded DNA intermediate that formed in replication fork regression, homology-dependent repair and mitosis, performing a significant role in genomic stability. Failure to remove HJ can induce an acceptable replication fork stalling and DNA damage in normal cells, leading to a serious chromosomal aberration and even cell death in HJ nuclease-deficient tumor cells. Thus, HJ is becoming an attractive target in cancer therapy. However, the development of HJ-targeting ligand faces great challenges because of flexile cavities on the center of HJs. This review introduces the discovery history of HJ, elucidates the formation and dissociation procedures of HJ in corresponding bio-events, emphasizes the importance of prompt HJ-removing in genome stability, and summarizes recent advances in HJ-based ligand discovery. Our review indicate that target HJ is a promising approach in oncotherapy.

## 1. Introduction

**Figure 1:**
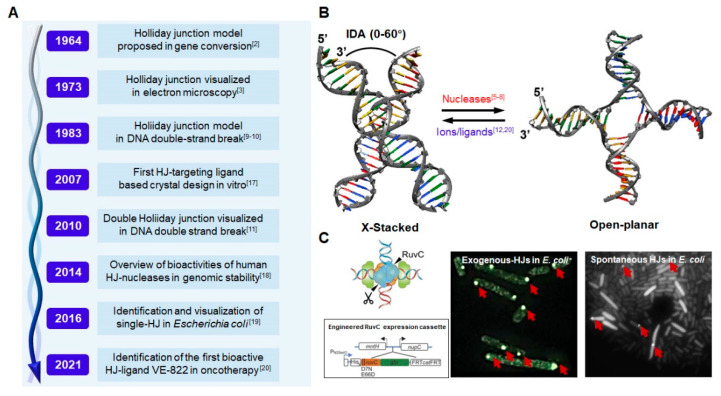
History, structure, and visualization of HJ. (**A**) Representative studies of HJ from 1964 to 2021. [2,3,9,10,11,17,18,19,20] (**B**) Schematic of two major HJ configurations termed “X-stacked” and “open-planar”, the X-stacked conformation usually exists in the condition of high concentration counter-ion (Mg^2+^) or certain ligand, and this conformation can be converted to the “open-planar” under the process of HJ nucleases [12]. (**C**) The visualization of HJ via an engineered RuvC protein (blue triangles), which defects nuclease activity and selectively targets HJ (**left**). The fluorescent foci represent the exogenous plasmid HJ (**middle**) and spontaneous HJ in *E. coli* (**right**) [19].

DNA Holliday junction (HJ) is a four-way nucleotide strand linking two duplex-DNA structures, and was discovered more than 60 years ago (Figure 1A) [1]. Initially, HJ was proposed by Robin Holliday to explain the DNA-strand exchange mechanism in gene-conversion events [2]. In the 1970s, HJ was first observed via electron microscopy in *Escherichia coli* (*E. coli*), providing conclusive evidence for the existence of HJ in organisms [3]. Subsequently, multiple nucleases for HJ resolution have been identified both in *E. coli* and human beings [4,5,6,7,8]. In the 1980s, the well-known HJ model in homologous recombination repair (HRR) initiated by DNA double-strand breaks (DSBs) was proposed, as well as the probable mechanism of two adjacent HJs, termed dHJs in meiotic recombination (detailed repair procedure shown in Section 2.1) [9,10]. However, in the 2010s, the HJ structure was actually visualized in eukaryotic cells and was proven to be an intermediate in homology recombination [11].

As a transient DNA intermediate, HJ performs its biological function mainly relying on conformational variation [12]. Generally, HJ generally has two configurations: “X-stacked” and “open-planar” (Figure 1B) [13,14,15]. In the presence of low salt concentrations, the “open-planar” state is a predominant configuration; otherwise, the junction can be transformed to a symmetric X-shape termed “X-stacked” at higher ionic concentrations. In addition, some junction resolvases destabilize these intermediates and convert their conformations from “X-stacked” to “open-planar” by cleaving base pairs and changing the interduplex angle (IDA) of arms. In addition, an increasing number of intermediate configurations of HJs have been captured using new spectroscopic, calorimetric, and single-molecule detection techniques [12,16]. An in-depth study revealed that the conformational changes of HJ depend on the electrostatic interactions of negatively charged chains and salt ions, and these changes have been suggested to play important roles in enzyme recognition and genetic recombination [12].

In recent years, bioactivity research on HJ has mainly focused on the consequence of HJ resolution in gene replication and DNA damage repair [19,20,21,22,23]. In eukaryotic cells, the DNA-HJ is strictly regulated by several nucleases; proper resolution maintains genome stability and ensures cell survival, and impaired resolution perturbs DNA replication and induces chromosomal instability and mitotic collapse [24,25,26]. However, the resolution mechanism and subsequent biological studies of HJ remain unclear at the single-molecule level, which is limited due to the deficiency of the HJ-identification tools. Fortunately, some scholars have realized the significance of the identification of intracellular HJs in situ. Xia presented a novel method to trap, map and quantify bacterial HJs using an engineered RuvC protein, fused with a green fluorescent protein (GFP), to identify intracellular HJs [19]. As shown in Figure 1C, both exogenous and spontaneous HJs can be visualized in single living cells (green fluorescent foci). Unfortunately, this approach is not suitable for HJ imaging in eukaryotic cells because of the complicated genome environment and species differences [27].

Despite these difficulties, fundamental and applied research on HJ has made great progress. As a functional nucleic acid, HJ has been widely utilized in various fields, including nanotechnology, drug design and delivery [28,29,30]. Among them, most efforts have been focused on the design and development of HJ-targeting ligands for antineoplastic therapy [31]. HJ is considered a potential participant in tumorigenesis and therapeutics because the location of HJ is theoretically overlapped with the broken duplex DNA across the genome. In human cells, tumor-associated mutations are likely located at the repeated sequences, as well as DNA breakage frequently occurs at repeated sequences in oncogenes [32,33]. In addition, HJ prefers to act as an obstruction to induce damage during gene replication or transcription processes, triggering the DNA damage response (DDR) and repair [34]. Thus, the HJ-targeting ligand may make tumor cells more sensitive to DNA damage agents considering that cancer cells exhibit broad repair deficiency [35,36].

## 2. Regulatory Mechanism of the DNA Holliday Junction in Human Cells

**Figure 2:**
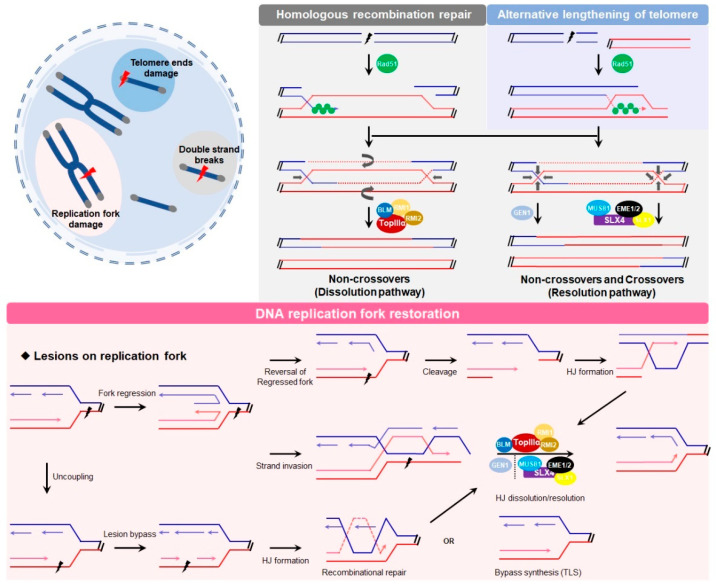
HJ-mediated repairs and the corresponding models. In HR and ALT pathway, the repair factor Rad51 binds one damage strand to initiate template search and strand invasion, resulting in the formation of single and double HJs, and these intermediates can be dissolved or resolved via different nucleases. In course of replication fork restoration, replication fork can undergo regression to form a single HJ when damage occurs, the end chain of single HJ invades into template to create a new HJ and then be removed by HJ nucleases. Besides, the regression may be reversed by BLM helicase, and the impaired fork may be cleaved to generate a single DSB for further recombinational repair. Again, the damage may be bypassed due to the discontinuous synthesis of DNA and eventually become a single-strand gap, which can be repaired by bypass synthesis or recombinational gap-repair.

There are many types of DNA damage, and the damage frequently occurs in various biological processes under the pressure of environments or chemical molecules (Figure 2) [37]. Every human cell suffers from serious DSB damage more than 50 times in response to replication arrest and telomere collapse throughout life [38,39]. However, this damage can be precisely repaired following the procedure of a recombination-mediated pathway between the undamaged sister chromatid, and sometimes between the homologous chromosomes. In these cases, intermediate HJs are produced and resolved step-by-step to generate proper repair products.

### 2.1. DNA Holliday Junction in Double-Strand Breaks and the Corresponding Dissociation Pathways

DSB is the first bioprocess for which DNA-HJ has been proven to exist [11]. When DSB occurs, the broken DNA is first cleaved via exonucleases to generate a 3′-overhang and then unwound to a 3′-single strand DNA. Next, the core protein Rad51 and other recombinases, including replication protein A (RPA) and Rad54, are recruited to the 3′-tails to form DNA-protein filaments and promote the search for the homologous template, which is termed single-strand invasion (SSI) [40,41,42]. Then, the invasion strand initiates base pairing, facilitating DNA strand exchange to form a single joint molecule known as a single HJ, and two adjacent single HJs form a dHJ. Subsequently, these HJs are dissolved into noncrossover and crossover products. As shown in Figure 2, there are three alternative pathways for HJ dissociation in human cells [43,44,45,46].

One approach is known as the dissolution pathway. The dissolution process involves Bloom’s syndrome helicase (BLM), Topoisomerase IIIα (TopIIIα), Rmi1 (RMI1) and Rmi2 (RMI2) proteins, which primarily dissolve dHJs and generate noncrossover products [46]. In this process, dHJ is disintegrated to a hemi-catenane intermediate and then separated into duplexes [47,48,49]. Among them, TopIIIα performs an important initiator role, which first forms an annular structure and induces a transitory gap in one DNA strand, allowing the second strand to be rotated through the gap to relieve the supercoiled topologies [50,51]. Then, the binding proteins RMI1 and RMI2 are recruited to the oligonucleotide binding domain of TopIIIα to enhance the dissolution of dHJs, and RRM2 acts as a stabilizer in the maintenance of the BTRR complex [22]. Finally, the BLM helicase is recruited to the 3′-tails of DNA gaps to unwind the hemi-catenane intermediate into DNA duplexes [52]. All components in this pathway seem to perform indispensable roles in HJ dissolution, and any loss or mutations may result in serious genomic instability [53,54,55]. Interestingly, harmful hyperactivation recombination repair is frequently observed in dissolvase-deficient cells, indicating that the dissolution pathway can be substituted for other approaches, but this replacement is harmful in cells [56].

The second approach utilizes structure-selective nucleases involved SLX1, SLX4, MUS81 and EME1 proteins to resolve single-HJ to produce crossover or noncrossover products. This process is also termed the resolution pathway. All endonucleases in this approach cleave the specific DNA topological structure, not the certain sequence. SLX1 is a conserved member in GIY-YIG superfamily nucleases and is generally inactive alone, and SLX4 is a scaffold protein that activates the activity of SLX1 via its C-terminal domain [57]. Research suggested that SLX1 and SLX4 could unite with each other to cleave several nucleic acid intermediates, including branched DNA, HJ and 5′-flap DNA [58,59,60]. In addition, SLX1 and SLX4 can cleave either the 5′- or 3′-sides of the single or duplex-strand DNA around the branch region, suggesting that the SLX complex may cleave the DNA-HJ at multiple sites [58]. MUS81 is a homology protein of XPF-ERCC1 endonuclease and dimerizes with EME1 or EME2 to perform nuclease activity [61]. MUS81 and EME1 have a similar nuclease domain and two repeat helix-hairpin-helix (HhH) domains. The DNA-HJ can bend into the HhH domain and expose the cleavage sites, but the cleavage induced by MUS81-EME1 only occurs adjacent to the 5′-junctions in the resolution process [62]. In addition, a recent report indicated that the MUS81-EME2 complex possessed better cleavage activity at HJ resolution than MUS81-EME1 in vitro, but a subsequent study revealed that the 5′-phosphated of terminus in DNA gaps limited nuclease activity in cells [63]. 

A third approach involves the GEN1 nuclease, which also belongs to the HJ resolution pathway. GEN1 is a member of the FEN/XPG family, which has four members: XPG/Rad2/ERCC5, FEN1, EXO1 and GEN1. Interestingly, these conserved proteins exhibit diverse substrate preferences despite sharing analogous cores, and only GEN1 recognizes and resolves HJ both in vitro and in vivo. In contrast to the MUS81-EME1 complex, dimeric GEN1 preferentially cleaves intact four-way junction by introducing symmetrical incisions across the junction [5]. Structural analysis reveals that the chromodomain of GEN1 may perform a crucial role in maintaining catalytic activity [64]. In addition, GEN1 shows a weak sequence specificity to guanines in the T-rich region, indicating that GEN1 has a broad sequence specificity to resolve various DNA junctions [65].

### 2.2. DNA Holliday Junction in Alternative Lengthening of Telomeres

Telomeres are composed of highly repetitive sequences (TAAGGG)_n_ bound with shelterin proteins to cap the ends of chromosomes [66]. The length of telomeres is decreased at a steady rate due to the end replication problem, as a reduction beyond the “Hayflick limit” will induce genetic loss, tumorigenesis and aging [67,68]. Research indicated that the telomere replication blockage could eventually become a telomeric DNA double strand break, which suggested that HJ might be formed in the telomere region [69]. In human cells, approximately 85% of tumor cells maintain telomere length via telomerase restoration [70]. An additional 10 to 15% of tumor cells activate the alternative lengthening of telomeres (ALT), a homology-directed repair, to prolong telomere length [71]. In the ALT pathway, the 3′-DNA overhang generated from exonucleases (e.g., MRE11) invades homologous telomeric DNA to form a DNA displacement loop as a Holliday junction (Figure 2). To date, several telomeric-HJ crystal structures have been presented in vitro, and the Holliday junction recognition protein (HJURP) is increasingly located at telomere dysfunction-induced foci (TIFs) in addition to HJ ligand, suggesting that HJs might be probably formed at intracellular telomeres [20,72,73].

In telomere repair, the BLM helicase is considered a central factor, and several repair proteins, such as FANCM and Rad54, have been reported to improve the activity of the BLM-mediated pathway on telomere DNA synthesis [74]. On the one hand, BLM is frequently recruited to telomeres and colocalizes with telomere damage foci [75]. On the other hand, BS patient cells show decreased ALT-associated PML bodies (APBs) and telomere shortening in contrast to BLM-overexpressing cells, and active BLM can decrease HJ-induced damage foci at telomeres, suggesting that BLM and associated repair proteins may have a major effect on telomeric HJ dissolution [20,76]. In addition, an antagonistic effect has been reported between the BLM-associated and SMX-associated pathways (SLX1-SLX4, MUS81-EME1 and XPF-ERCC1) on telomere regulation [77]. A recent study revealed that the SLX4-interacting protein, SLX4IP, inhibited the transcription of the BLM gene to reduce the telomere recombination level, and active BLM rescued the APB activity inhibited by the SLX4/SLX4IP complex [78]. The above results suggest that each regulatory pathway of telomeric HJ may have a negative effect on others, and the detailed orientation needs to be further defined.

### 2.3. DNA Holliday Junction in Replication Fork Restart

DNA replication is an exactly controlled biological event that maintains genomic stability and avoids tumorigenesis. The replication fork can be arrested when encountering any obstructions in the form of damage, protein and secondary structure [79]. Once the obstacle is removed, the stalled replication fork will restart again; otherwise, the fork can be stalled or collapsed to generate a broken strand for further repair. HJ is regarded as a potential intermediate in replication fork restoration, and its regulatory mechanism has been discussed in detail by Sarbajna and Petermann [18,80].

In replication fork restoration, the assembly process of DNA junctions depends on the location of damage. As shown in Figure 2, the single-strand DNA lesion may induce fork regression or replication restoration. In the former event, the replication fork is regressed and paired with a newly synthesized strand to form a single HJ named “chicken foot”, and which can theoretically be reversed by BLM helicase. Then, the unsolved lesion may be cleaved to generate a one-ended DSB, and this damage can be processed into a canonical HJ for further repair. In the latter event, the 3′-DNA overhang invades the DNA template and forms a dHJ, and all these intermediates are finally removed via the dissolution or resolution pathways. In the third way, the synthesized DNA can be functional unwound and the single-strand DNA lesion may be temporarily bypassed due to the discontinuously synthesis of DNA. Subsequently, these gaps may be removed by the BTR, SLX-MUS81 and GEN pathways or bypass synthesis (TLS).

At present, the physical state of HJ in the replicating fork is still controversial. One persuasive piece of evidence is that the recombination factor Rad51 can be recruited to the stalled replication fork to initiate fork restart in a short time (2 h) [81]. As Rad51 promotes homology research and catalyzes the formation of HJs, this junction structure is considered the most suitable intermediate for fork restoration. In addition, BLM helicase can be subsequently recruited to the stalling site after 2–6 h, and the deficiency of BLM accelerates fork collapse and prolongs Rad51 foci at the damage site, indicating that the BLM-mediated pathway is positively involved in recombination initiation during replication [80].

## 3. Biological Effects Induced by Unsolved DNA Holliday Junctions in Cells

As mentioned above, four-way DNA-HJ is an essential intermediate for the repair of double-strand breakage and replication fork stalling. Proper regulation of HJ is important for genomic stability and cellular survival. In normal cells, the precise cooperation of nucleases removes HJs to avoid damage. In contrast, the deficiency of HJ nucleases can generate unresolved intermediates and lead to chromosome aberrations. Herein, some major damage events induced by unsolved HJs are introduced.

### 3.1. Replication Fork Impairment and Chromosome Aberration

**Figure 3:**
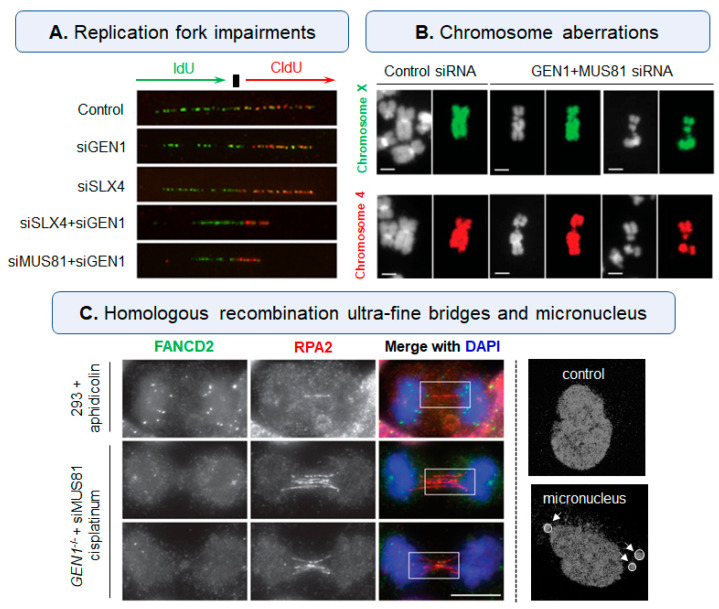
Typical damage caused by unsolved-HJ in nucleases-deficient cells. (**A**) Combined depletion of SLX4-GEN1 or MUS81-GEN1 slowed the progression of replication fork using DNA fiber assay, fibers were stained with IdU (green) and CldU (red) antibodies to represent the replication fork progress. (**B**) Combined depletion of MUS81-GEN1 in BS patient cells induced serious chromosome breakages (sained with DAPI in chromosomes X and 4). (**C**) Combined depletion of MUS81-GEN1 increased homologous recombination ultra-fine bridges (HR-UFBs) in cisplatinum-treated cells compared with normal 293 cells treated with aphidicolin (HR-UFBs were stained with RPA2 and FANCD2), and HJ-ligand induced micronucleus in cancer cells [20,82,84,86].

Because the single-molecule HJ cannot be visualized in cells right now, its potential bioactivity is indirectly verified via associated nucleases. As HJ is frequently assembled under the replication fork stalling, the biological effect of unsolved HJ needs to be detailed described in mitosis. As shown in Figure 3A, the length of DNA fibers is not affected in siGEN1 or siSLX4 single treatment group, indicating the deficiency of single nuclease generates a slight impairment but does not delay the progression of DNA replication. In addition, the lengths both in IdU (green) and CldU (red) tracts are significantly decreased upon the combined depletion of nucleases, which belong to different HJ-resolution pathways. These results indicate that some HJ nucleases (SLX-MUS and GEN1) may be functionally redundant with each other to guarantee the removal of HJ, and the unsolved HJ might slow the progression of replication fork [82,83,84]. Again, the combined depletion rather than single depletion of MUS81/GEN1 or SLX1/GEN1 in Bloom’s Syndrome (BS) patient cells can break the intact replication fork, leading to improper chromosome segregation and S-phase arrest [81]. In addition, MUS81-depleted cells show normal progression in DNA replication but lead to serious replication fork collapse under increasing exogenous pressure. Hence, the unsolved HJs should be controlled to a certain degree; otherwise, they will induce serious replication defects and even cell death. As replication fork damage eventually leads to chromosomal aberration, chromosomes frequently generate several condensed and uncondensed regions in HJ nuclease-depleted cells, and these structural aberrations do not arise from broken chromosomes or translocation events, indicating that the uncondensed chromosomes are derived from unresolved intermediates (Figure 3B) [85].

### 3.2. Ultrafine Anaphase Bridges and Mis-Segregation

The dissociation of HJ is important for chromosome segregation, which depends on the proper removal of nucleic acid connections between sister chromatids. To investigate the regulation of HJ on chromosome division, a homologous recombination ultrafine bridge formed with unsolved HJ was identified and termed HR-UFB in mitosis [86]. HR-UFB cannot be stained by conventional DNA dyes but can be visualized by its binding proteins (RPA or other repair proteins) using an immunofluorescence assay [87,88]. In HJ nuclease-depleted cells, unresolved HJs cannot trigger cell cycle checkpoints and maintain the bridges to anaphase, but the bridges in normal cells are conversely broken before cell division. (Figure 3C, left). In brief, persistent HR-UFBs can transmit damage from early mitosis to the next generation and even induce the nuclear fusion or death of daughter cells. As a consequence, there are many mis-segregation events and chromosome fusions in HJ nuclease-deficient cells, and these malignant incidents can be repaired via the removal of unsolved DNA junctions [86]. Thus, the unsolved HJs can generate continuous ultrafine anaphase bridges to induce DNA damage and segregation error during mitosis.

### 3.3. Irregular and Catastrophic Nuclei

Micronucleus is the manifestation of chromosomal aberration in interphase cells. All BLM-, SLX1-MUS81- and GEN1-depleted cells could enhance the accumulation of DNA damage from consecutive mitosis, gradually resulting in irregular and catastrophic nuclei (Figure 3C, right) [18]. In addition, the micronucleus level upon the combined-treatment of siSLX4 and siGEN1 (SLX4 and GEN1 small interference RNA) is slightly higher than the single-treatment in cells, and a higher increase of the micronucleus level is observed in addition to cisplatin or HJ-ligand [18,20,84]. Hence, these results indicate that the silencing of nucleases can gradually suppress the dissociation of HJ to induce incremental DNA damage and that chemical molecules have stronger effects on HJ formation and nuclear collapse.

## 4. Holliday Junction-Targeting Anticancer Ligands

DNA agents have extensive applications in imaging, diagnosis and antitumor research. The most well-known aspect of DNA agents is their development as chemotherapy drugs in oncotherapy. However, nonselective DNA-binding chemotherapy ligands are limited to application in clinical treatment due to their serious cytotoxicity. Hence, some novel nucleic acid structures, including G-quadruplexes, three-way junctions and four-way junctions, have become attractive in medicament design and development [89,90,91]. The molecule targeting the DNA junction was first proposed by N.R. Kallenbach and has been demonstrated to have a potential therapeutic application [92]. HJ is the most famous four-way junction, which is preferred during DNA damage repair. Thus, the HJ is probably formed in an event-driven process, not in a sequence-specific manner, and this case increases the challenge of ligand design and synthesis. In brief, the development of ligands targeting HJ is only an emerging field.

**Figure 4:**
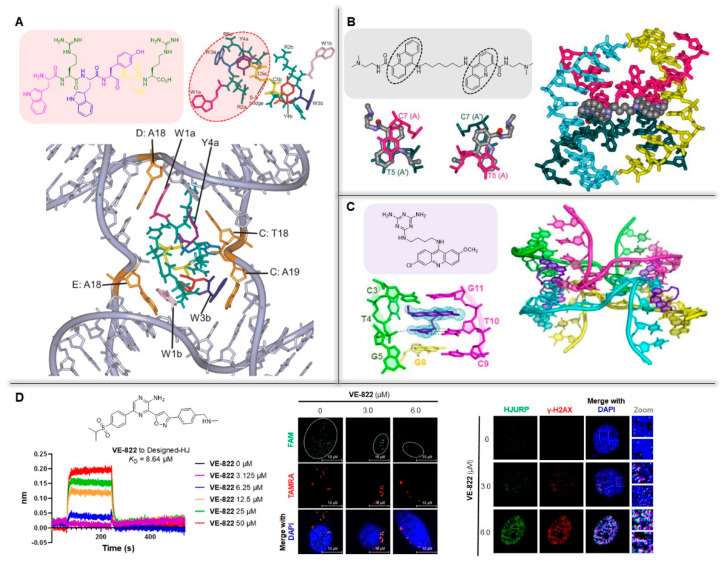
Structures, binding modes and bioactivities of HJ ligands. (**A**) Monomeric and dimeric structures of hexapeptide WRWYCR and its predicted binding model with HJ [94]. (**B**) Chemical structure of dimeric acridine C6, along with the crystal structure with HJ (PDB: 2GWA) [17]. (**C**) Chemical structure of acridine derivative Z1 and its crystal structure with HJ (PDB: 6M5J) [97]. (**D**) Structure of HJ-ligand VE-822 and its binding affinity to HJ using Bio-Layer Interferometry assay (**left**), and VE-822 promote the assembly of transfected HJ in cells using fluorescence quenching assay (**middle**) and induce HJ-stabilized damage (**right**) [20].

The development of HJ ligand was initially aimed at resolving the HR governs in genetics and repair. Some HJ-binding hexapeptides (KWWCRW and WRWYCR) were first discovered and demonstrated to inhibit various nucleases and induce HJ accumulation [93,94]. Structural analysis reveals that the peptide WRWYCR (the same below) is dimerized through a disulfide bridge and bound to the central cavity of DNA-HJ with a high binding affinity (*K*_D_ = 14 nM), which is approximately 4- to 9-fold higher than that of the forked DNA (Figure 4A). In addition, the peptide is demonstrated to be a competitive inhibitor of HJ nucleases and preferentially bound to the “open-planar” configuration via DNA aromatic stacking interactions (Figure 4A) [95]. Subsequent biological studies indicate that the peptide WRWYCR induces slight proliferation arrest in several tumor cells (A549, Du145, LnCAP, PPC-1, DuPro-1 and PC3) with IC_50_ values over 100 μM, and the increasing damage foci (γ-H2AX and 53BP1) indirectly indicates that the peptide may target HJ in cells [96]. In contrast to the high HJ-binding affinity, the weak cytotoxicity of the peptide suggests that HJ blockage may not be a lethal event in cells. In addition, more than fifty percent of PC3 cells are arrested in S-phase upon treatment with peptide, and combined treatment with peptide and chemotherapeutic drugs acting in S-phase (doxorubicin and HU) exerts an additive effect against cancer cells. In contrast, docetaxel acting in M-phase does not have a synergistic effect with peptide, suggesting that the HJ-targeting agent may act as a potential chemosensitizer in antitumor treatment [94].

On the other hand, the development of HJ-targeting chemical molecules also faces great challenges. The overall C2 symmetry and larger binding pocket (25 × 10 Å) compared with canonical duplex DNA increase additional difficulties in ligand design and synthesis. Acridine-based molecules have been known as classical DNA agents by inserting themselves into Watson-Crick base pairs, and the original design of the HJ-ligand is derived from acridine derivatives [98,99]. In 2007, Brogden designed alkyl chain-linked dimers of 9-aminoacridine-4-carboxamide as the first four-way junction ligand. As shown in Figure 4B, crystal analysis indicates that the compound binds across the junction with the acridine aromatic rings bound in an intercalative fashion [17]. Subsequently, Chien presented a novel HJ-acridine crystal complex and found that triaminotriazine-acridine (Z1) could efficiently bind T: T mismatched DNA sequences and promote the assembly of four-way DNA junctions [97]. Further study reveals that compound Z1 specifically recognizes the intercalation region of the CTG repeat at a 2:1 stoichiometry to induce thymine-base rolling, allowing the DNA backbone to bend to form a noncanonical four-way junction (Figure 4C). It is regrettable that the bioactivities of these acridine derivatives have not been studied in depth, but the above results reveal that the HJ-targeting ligand can be accurately designed by allowing the suitable distance of acridine to probe the central space of the four-way junction.

In addition, the bioactive molecule VE-822 was screened from a small DNA damage agent library and identified as a novel HJ ligand in recent research. In interaction study shown in Figure 4D (left), the binding curve closely reached saturation with the increasing concentration of compound, and the disassociation constant (*K*_D_) value of VE-822 to HJ was determined as 8.64 μM [20]. Furthermore, the effect of VE-822 on HJ assembly was detected using fluorescence quenching assay. In this assay, two adjacent sub-chains of HJ were labeled with fluorophore carboxylfluorescein (FAM, green) and quencher tetramethylrhodamine (TAMRA, red), respectively, and the fluorescence of FAM was significantly decreased, indicating VE-822 could promote the assembly of transfected DNA-HJ in cells (Figure 4D, middle). Cell biological evaluations suggest that VE-822 can inhibit HR-based repair to induce extensive DNA damage in osteosarcoma U2OS cells, and this damage can be restored by overexpressing the HJ-dissolution enzyme BLM. Most importantly, increasing colocalization foci of γ-H2AX and the Holliday Junction Recognition Protein (HJURP) are observed in a dose-dependent manner, suggesting that the damage induced by VE-822 is closely linked with the stabilized HJs (Figure 4D, right). A subsequent cell signaling study reveals that the damage caused by VE-822 is blocked in DNA-PKCS-silenced cells (via DNA-PKCSi Nu7026 and RNAi), indicating that the activation of DNA-PKCS may be an important sensor in response to HJ-stabilized damage. In oncotherapy, VE-822 exhibits a synergistic effect with the DNA damaging agent doxorubicin as well as the hexapeptide WRWYCR mentioned above, and a low concentration of VE-822 obviously enhances the antitumor effect of doxorubicin in tumor cells, which can be mitigated by the overexpressed BLM. Taken together, HJ has considerable promise as a sensitizing target in anticancer therapy. 

## 5. Conclusions and Prospect

The HJ model has been proposed for over half a century, and the importance of this four-way DNA junction is attracting more attention from researchers. Imaging studies confirm that HJ indeed acts as a high-order nucleic acid intermediate, not a theoretical model in genetic recombination. In vitro crystallography and molecular dynamics simulation studies introduce the structural conversion and thermodynamic stability of HJ configurations in detail, providing a theoretical basis for the metabolism of DNA-HJ in cells. Subsequently, several major HJ machining processes involving BLM-topoisomerase IIIα-RMI1-RMI2, SLX1-SLX4-MUS81-EME1 and GEN1 have been identified to decompose HJs into crossover or noncrossover products. Failure to process HJs generally induces replication fork collapse, chromosome segregation aberrance and even cell death. Undoubtedly, a proper HJ process is a necessary defender to maintain genomic stability and avoid tumorigenesis. In contrast to other higher-order nucleic acid structures, HJ is an instantaneous intermediate randomly formed in the whole genome. The dissolution and the bioeffect of single-molecule HJ have not been completely defined, because the existing studies on HJs are still restricted to the whole cellular effect. Hence, the visualization and regulatory mechanism of the single-molecule HJ is of great significance for further ligand design and functional studies.

HJ is an ideal druggable target with a central cavity to accommodate suitable ligands, and the stabilization of DNA junctions is prone to induce DNA damage in repair-deficient cancer cells. Therefore, the discovery of HJ-targeting agents has an exciting future in oncotherapy. To date, some alkyl-chain-linked acridine derivatives exhibit the potential for the development of further HJ-targeting ligands. Noticeably, the conformation factors cannot be ignored in HJ-based drug design, as “X-stacked” and “open-planar” HJs possess entirely different cavities. One example is that a certain flexible molecule may bind the groove to convert the conformation of HJ. In brief, there are few studies about HJ-ligand interactions. The discovery of active molecules is the most urgent aim right now, and the structure-activity relationships (SAR) need to be analyzed in detail to illustrate the active sites of HJ structures for further drug design.

## Data Availability

Not applicable.
